# Angiographic Evaluation of the Feeding Artery in Skull Base Meningioma

**DOI:** 10.3390/jcm12247717

**Published:** 2023-12-15

**Authors:** Hironori Arima, Yusuke Watanabe, Yuta Tanoue, Hiroki Morisako, Taichiro Kawakami, Tsutomu Ichinose, Takeo Goto

**Affiliations:** 1 Department of Neurosurgery, Osaka Metropolitan University, Osaka 545-8585, Japanhmorisako@omu.ac.jp (H.M.); gotot@omu.ac.jp (T.G.); 2 Department of Neurosurgery, Tsukazaki Hospital, Himeji 671-1227, Japan

**Keywords:** skull base, meningioma, feeding artery

## Abstract

To identify the characteristics of feeding arteries in skull base meningioma including location and prevalence, we evaluated the distributions and types of feeding arteries in skull base meningioma by cerebral angiography and assessed relationships to tumor attachment. We enrolled patients with skull base meningioma who underwent MRI and cerebral digital subtraction angiography (DSA), from September 2015 to October 2022. Subjects comprised 115 patients (32 males, 83 females; mean age, 52.7) with 117 meningiomas, showing tumor attachments around the “cavernous sinus to the upper part of the clivus” (Area 1), “lower part of the clivus to foramen magnum” (Area 2), and “tentorium around the petrous bone” (Area 3). Frequent arteries, such as the dorsal meningeal artery (DMA), the ascending pharyngeal artery (APA), the tentorial artery (TA), and the petrosal branch (PB) of the middle meningeal artery (MMA) were analyzed in terms of their associations with tumor attachment to Areas 1–3. Meningiomas with the DMA as a feeding artery correlated with tumor attachment to Area 1 (*p* < 0.001). Meningiomas with the APA correlated with tumor attachment to Area 2 (*p* < 0.001). Meningiomas with the TA correlated with tumor attachment to Area 3 (*p* < 0.001). The PB correlated with Area 3 (*p* < 0.05). Our study founded that visualization of these arteries correlated well with specific areas. These arteries were also the main feeders in each type of skull base meningioma.

## 1. Introduction

Although various approaches to skull base meningioma are advocated [1,2,3,4,5], resection of it is considered challenging due to its deep location and the presence of important surrounding structures. Sufficient internal decompression is always essential for safe tumor resection, but such procedure often causes bleeding from the tumor, especially in vascular-rich tumors. Particularly in skull base meningioma, the feeding artery often comes from a deeper area of the skull base, so a proper surgical approach to attacking the feeding arteries is essential. Moreover, resection of feeders themselves also provides more mobile tumors, in addition to tumor devascularization [2]. Thus, in skull base meningioma, anatomical understanding of the feeding arteries is very important not only for diagnosis but also for its treatment. 

Although some studies have described dural vessels in normal subjects [6,7], few reports have provided detailed information regarding feeding arteries in meningioma patients [2], and even those articles regarding embolization for meningioma have contained little information about the anatomical characteristics and distributions of feeding arteries in meningioma [8,9,10,11]. In recent years, some studies have pointed out the possibility of the feeding artery as an anatomical land mark of skull base meningioma [2,4], but these tumors are relatively rare so it is usually difficult to evaluate them in large numbers. This study retrospectively analyzed images from MRI and angiography of skull base meningiomas in a relatively large number and reported the detailed distributions and frequency of feeding arteries to demonstrate the relationship between tumor attachment and feeding arteries.

## 2. Materials and Methods

### 2.1. Study Population

We searched our institutional database for patients who underwent cerebral angiography for meningioma between September 2015 and October 2022. Contrast-enhanced T1-weighted MRI of each patient was checked, and those patients showing tumor attachment at the cavernous sinus, clivus, foramen magnum, petrous edge, or tentorium around the petrous bone were included in this study. Patients who did not undergo contrast-enhanced MRI were excluded from this study. Tumor attachments and feeding arteries in each meningioma were examined retrospectively. The institutional ethics committee approved this study, and written informed consent for the publication of images was obtained from all patients.

### 2.2. MRI and Angiography Protocols

**Figure 1:**
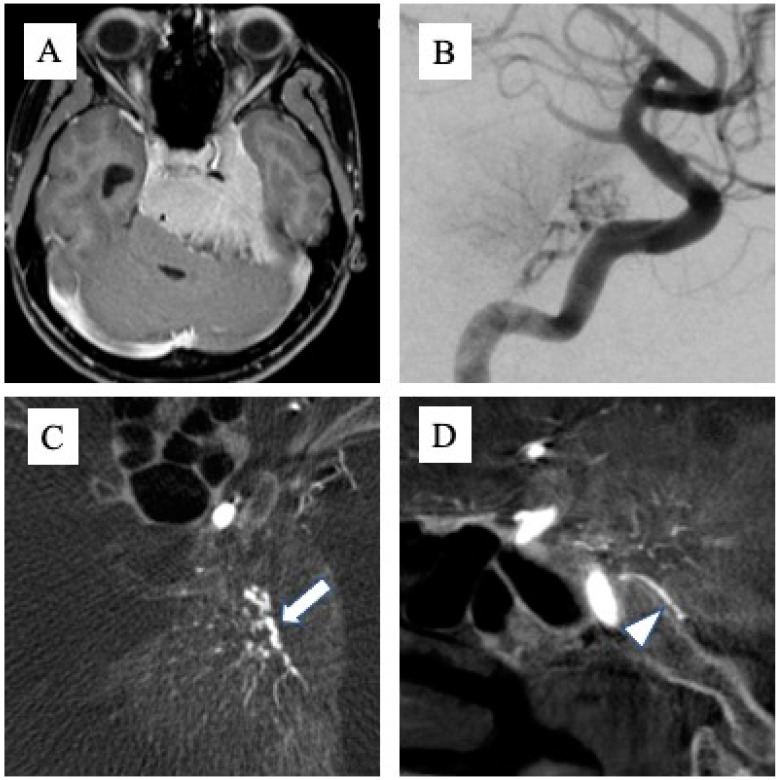
Cone-beam CT obtained from digital subtraction angiography. When the feeding artery was difficult to define via 2D angiography, cone-beam CT helped to determine the running course and accurately identify the vessel. (**A**) An example of a huge meningioma with broad attachment. (**B**) When various feeding arteries were visualized, it was difficult to define the accurate name of artery from 2D angiogram alone. (**C**,**D**) The anatomical relationships and running courses of the tentorial artery (C: arrow) and dorsal meningeal artery (D: arrowhead) were easy to understand from cone beam CT.

Tumor attachments were defined with reference to MRI T1-enhanced images (MAGNETOM Avanto 1.5T: TE/TR, 3.6/8 ms; number of excitations, 1; field of view, 220 × 220 mm; matrix size, 220; voxel size, 1.00 × 1.00 × 1.00 mm; inversion time (TI) 1100 ms, or MAGNETOM Vida 3.0T; TE/TR, 2.8/6.3 ms; number of excitations, 1; field of view, 220 × 220 mm; matrix size, 256; voxel size, 0.86 × 0.86 × 0.9 mm; inversion time (TI) 1100 ms, Siemens Healthcare GmbH, Forchheim, Germany), and the part with a clear mass more than 2 mm thick and with obvious MRI enhancement was defined as tumor attachment. The presence or absence of attachment to Areas 1, 2, and 3 was determined for each tumor, and if a wide range of attachments was seen across multiple areas, these were counted as duplicates. The feeding artery was determined from cerebral angiography (angiography system: Artis zee biplane; Siemens Healthcare GmbH, Munich, Germany). Digital subtraction angiography was performed using a 5-Fr catheter, with 8 mL of contrast medium at a rate of 6 mL/s for the common carotid artery (CCA), 6 mL of contrast medium at a rate of 4 mL/s for the internal carotid artery (ICA), and 5 mL of contrast medium at a rate of 3 mL/s for the external carotid artery (ECA) and the vertebral artery (VA). An acquisition technique DSA was as follows: voltage,73 kV; Current, Automatic mA; pulse width, 100 ms; acquisition, variable frame rate 4-2-1 frame/s; 4 frame/s 4 s, 2 frame/s 4 s, and 1 frame/s after that; injection delay, 1 s. Angiography from the CCA, ICA, or ECA was performed ipsilateral to the tumor in all cases. When tumor attachment was around the foramen magnum, angiograms were obtained from bilateral VAs. Contralaterally, an angiogram from the CCA was obtained first. If tumor staining was evident, angiography was added from the ICA or ECA to clearly visualize the feeding artery. In addition, cone-beam computed tomography (CBCT) from the ipsilateral CCA was taken in all cases, with 56 mL of a double-diluted contrast medium injected at 2 mL/s. An acquisition technique CBCT was as follows: total projection image, 496; total scanning angle, 200°; acquisition time, 19 s; detector size, 48 cm (diagonal); matrix size, 1240 × 960; voltage, 70 kV; current, Automatic mA; pulse width, 12.5 ms.When determining the feeding artery using DSA alone proved difficult, we referred to anatomical information that could be confirmed with CBCT, such as the running course of the artery (Figure 1A–D). Two endovascular specialists checked DSA and CBCT and defined the feeding artery of the tumor.

### 2.3. Subdivision of Areas of Tumor Attachment

**Figure 2:**
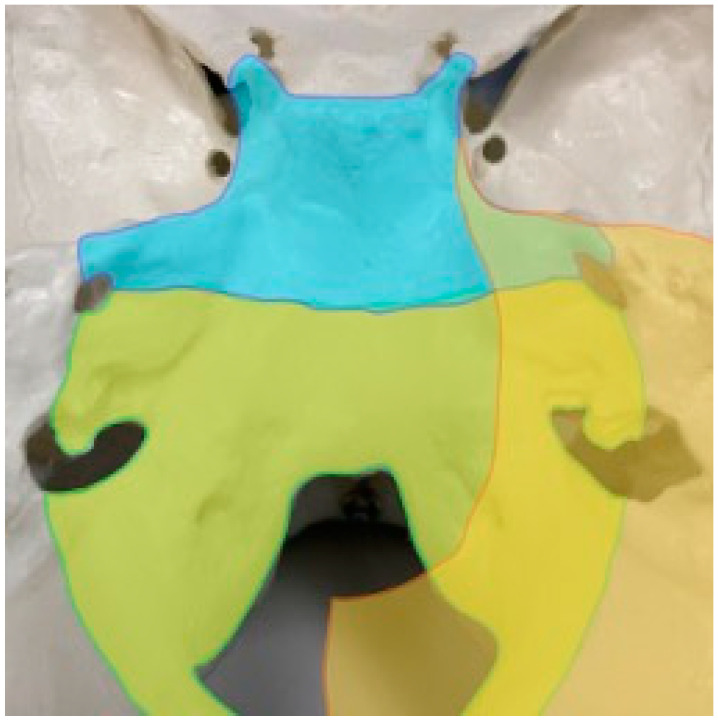
Defining the area of tumor attachment. The dura mater encompassing the cavernous sinus, clivus, foramen magnum, and tentorium is separated into three areas. Area 1 (blue): cavernous sinus and upper clivus up to the internal auditory meatus; Area 2 (green): lower clivus down to the internal auditory meatus and foramen magnum; Area 3 (yellow): tentorium cerebelli.

Attachments for meningioma are always continuous, so we intentionally divided the areas of tumor attachment into three areas. The cavernous sinus and the upper part of the clivus were defined as Area 1; the lower part of the clivus, as well as the area around the foramen magnum, were considered Area 2; and the tentorium around the petrous bone was defined as Area 3 (Figure 2). Referring to a report by Khaled et al. [12], the upper part of the clivus in this study means the rostral area of the clivus from the height of the internal auditory meatus, while the lower part of the clivus represents the caudal area from that height. We assessed the feeding arteries under this anatomical classification because different surgical approaches should be chosen to attack these regions in practice.

### 2.4. Statistical Analysis

The number of feeding arteries detected via angiography was counted, and frequent arteries, namely, the dorsal meningeal artery (DMA), the ascending pharyngeal artery (APA), the tentorial artery (TA), and the petrosal branch (PB) of the middle meningeal artery (MMA), were analyzed in terms of their relationships with tumor attachment in Areas 1–3. Univariate analyses using Pearson’s chi-square test and multivariate analysis using logistic regression analysis were used to evaluate the relationships. All statistical analyses were performed using JMP12 software (SAS Institute, Cary, NC, USA). Values of *p* < 0.05 were accepted as significant.

## 3. Results

A total of 168 meningioma patients underwent angiography in our institute between September 2015 and October 2022. Of these, 53 patients were excluded from the study because of the absence of tumor attachment in Areas 1–3. In total, 117 tumors in 115 patients showed attachment in Areas 1, 2, and/or 3, with multiple meningiomas in 2 patients. These 115 patients comprised 32 men and 83 women, with a mean age of 52.7 years (range, 22–84 years). Meningiomas with attachments in Areas 1, 2, and 3 were seen in 92, 50, and 67 of the 117 tumors, respectively, and meningiomas with extensive attachments were counted as duplicates.

### 3.1. Blood Vessels Visualized on DSA for Skull Base Meningioma

The key feeding arteries from the ICA were the TA, the DMA, and the posterior ethmoidal artery. The key feeding vessels from the ECA were the MMA, the accessory meningeal artery, the artery of the foramen rotundum, the sphenopalatine artery, the mastoid branch of the occipital artery, and the APA. Particularly with the MMA, angiograms showed the PB, the petrosquamous branch, the anterior branch, and the recurrent meningeal branch. The posterior meningeal artery and the tentorial artery arising from the posterior cerebral artery or the superior cerebellar artery were found as feeding arteries from the posterior circulation. As anatomical variations, two cases of TA originating from the recurrent meningeal branch of the MMA and one case of TA originating from the ophthalmic artery were observed, and both were classified as TA. The frequencies of feeding arteries are shown in Table 1.

### 3.2. Correspondence between Feeding Artery and Tumor Attachment

Arteries frequently visualized on DSA, namely, the DMA, APA, TA, and PB, were analyzed in terms of their relationships with tumor attachment to Areas 1, 2, and 3 (Table 2).

#### 3.2.1. Dorsal Meningeal Artery

**Figure 3:**
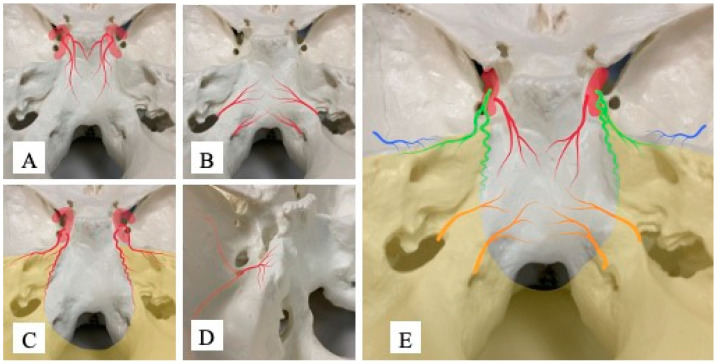
Schema of important arteries and distributions in skull base meningioma. (**A**) Distribution of the dorsal meningeal artery (DMA): This artery usually arises from the meningohypophyseal trunk and courses inferiorly along the dorsal side of the clivus and partially reaches the inferior part of the clivus. (**B**) Distribution of the ascending pharyngeal artery (APA): This artery usually arises from the external carotid artery and passes superiorly to distribute around the foramen magnum and lower clivus as the neuromeningeal trunk of the APA. (**C**) Distribution of the tentorial artery (TA): The medial branch usually arises from the meningohypophyseal trunk and the marginal branch arises from the inferior lateral trunk, passing posteriorly along the tentorium and distributing around the tentorium. (**D**) Distribution of the petrosal branch (PB): This artery arises from the middle meningeal artery at the foramen spinosum and passes medially to the petrous apex, where it is believed to be distributed around the petrous apex and part of the tentorium. The anterior and posterior branches of the MMA are shown in light red. (**E**) Summary of the four vessels: Red, DMA; orange, APA; green, TA; blue, PB.

The DMA was strongly associated with Area 1 (univariate analysis, *p* < 0.001; multivariate analysis, *p* < 0.001) and was considered an important feeding artery in Area 1 (Figure 3A). No significant associations were observed with Area 2 (*p* = 0.15) or Area 3 (*p* = 0.76). In addition, when the DMA was present, 97.8% (44/45) of tumors displayed attachments in Area 1. However, even in the absence of the DMA, 66.7% (48/72) of cases showed attachment in Area 1 (Table 3). The presence of the DMA was thus specific but not particularly sensitive for tumor attachment in Area 1.

#### 3.2.2. Ascending Pharyngeal Artery—Neuromeningeal Branch

The APA was strongly associated with Area 2 (univariate analysis, *p* < 0.001; multivariate analysis, *p* < 0.001) and represented an important feeding artery in Area 2 (Figure 3B). In addition, univariate analysis showed a significant association between the APA and Area 1 (*p* < 0.05) but no significant association with Area 3. When the APA was present, 90.2% of cases (37/41) displayed attachment to Area 2. When the APA was absent, only 17.1% (13/76) of cases showed attachment to Area 2 (Table 4). The presence of APA was thus sensitive and specific for Area 2 tumor attachment. When considering the relationship between the APA and Area 1, part of the clivus around the border of Areas 1 and 2 may be supplied by the APA.

#### 3.2.3. Tentorial Artery—Marginal and Medial Branches

The TA was strongly associated with Area 3 (*p* < 0.001) and formed an important feeding artery in Area 3 (Figure 3C). In addition, the TA showed a significant association with Area 1 in univariate analysis (*p* < 0.05), and part of the cavernous sinus in Area 1 may be supplied by the TA. No significant association was found with Area 2 (*p* = 0.81). When the TA was present, 85.4% (53/62) of cases showed attachment to Area 3. When the TA was not present, only 25.5% (14/55) of cases displayed attachment to Area 3 (Table 5). The TA was therefore found to be a sensitive and specific blood vessel in Area 3, and part of Area 1 may be supplied by the TA.

#### 3.2.4. Petrosal Branch of Middle Meningeal Artery

The PB was associated with Area 3 in multivariate analysis (*p* < 0.05), suggesting that the PB is a feeding artery associated with the tentorium. Considering its distribution, the PB was associated with tumor attachment in the tentorium along the petrosal bone (Figure 3D), whereas no significant associations with Areas 1 or 2 were observed. When the PB was present, 86.3% of cases (38/44) showed attachment to Area 3, but 74.0% (54/73) displayed attachment to Area 3 even when the PB was not present (Table 6). The PB was thus considered to be a specific feeding artery for Area 3 but did not appear to represent a sensitive blood vessel in this area.

A schematic image of feeding arteries for skull base meningioma based on angiographic information is shown in Figure 3E.

## 4. Discussion

Skull base meningioma is the most challenging surgery among meningiomas [1,13], so tumor devascularization and mobilization are more important for safer tumor resection. Since feeding arteries are considered to play a role not only in supplying blood flow but also in anchoring the tumor to the dura mater [2], the effective transection of these arteries makes the tumor bloodless and mobile. A correct understanding of the running course and origin of feeding arteries is thus essential to achieve safer, more efficient surgery. In an outstanding analysis of the distribution of dural blood vessels in a study of previously healthy cadavers [6], Martins et al. provided a large amount of information for understanding the normal distribution of blood flow to the dura. Although performing such a detailed evaluation with an angiogram is difficult, our study provides useful information for understanding the distribution of feeding arteries specifically in patients with skull base meningioma. Our analysis revealed the APA as a highly sensitive and specific blood vessel in the lower part of the clivus and around the foramen magnum, and the TA as a highly sensitive and specific blood vessel in the tentorium. We also found that the presence of the DMA was strongly associated with tumor attachment to the upper part of the clivus. The APA was also suggested to be associated with the boundaries of the upper and lower clivus, and the PB was associated with the part of the tentorium connected to the petrous edge (Figure 3, Table 6). These blood vessels are particularly major feeding arteries in skull base meningiomas and show clear associations with attachment area. Understanding these tendencies and attachments may offer substantial advantages when considering and handling feeding arteries of skull base meningioma before and/or during the operation. Particularly considering small corridor approaches, including endoscopic approaches, information about feeding arteries plays an important role in considering preoperative embolization or tailored approaches to prevent intraoperative bleeding.

### 4.1. Feeding Arteries in Skull Base Meningioma

The distributions of the TA, DMA, APA, and PB as described in the report by Martins et al. are very similar to the distributions found in our analysis. To be sure, there is a possibility of underestimating blood vessels that are too tiny to visualize on angiograms, but even if these unvisualized arteries provide some blood flow to Areas 1–3, the clinical significance of such feeding arteries would be much less than those of the DMA, APA, TA or PB. Our analyses reveal that the feeding arteries in skull base meningioma are very similar to the physiological distributions, so a strong visualization of a feeding artery that is usually less involved in the area might lead to the consideration of another disease with a high blood supply, such as a solitary fibrous tumor.

As described above, physiologically dominant blood vessels may tend to develop prior to or early in meningioma, resulting in a more important role in supplying the tumor compared to small minor vessels. Our analysis suggests that the main blood vessels playing an important role in meningioma with attachments around Areas 1–3 are the TA, DMA, APA, and PB. In our series, at least one of these vessels was visualized in all cases, without exception. Judging from these results, such knowledge is very important and useful information not only prior to surgery but also for angiography or preoperative embolization. The advantages of preoperative tumor embolization in meningiomas have been discussed in many reports [8,9,10,11,14,15], and the results of our study also support the efficacy of this option. Among all the arteries investigated in this article, the TA, DMA, APA, and PB potentially provide blood flow to the cranial nerves or form anastomosis with cerebral vessels [16,17]. Maximum care must therefore be taken when performing embolization from these arteries. A report and summary of safety and technical notes on embolization for skull base meningioma in our institute is now in progress.

Blood vessels such as the MMA, the recurrent meningeal artery, the accessory meningeal artery, and the AFR (as shown in Table 1) are occasionally depicted as feeding arteries, but these vessels were not originally thought to supply blood flow to Areas 1–3. This is believed to indicate an overestimation of feeding arteries, particularly for huge meningiomas with attachments not only in Areas 1–3 but to the anterior skull base, the middle skull base, or the sphenoid ridge. We must therefore pay attention to feeding arteries other than the DMA, APA, TA, and PB, especially when the attachment is extensive and broad.

### 4.2. Feeding Artery around the Cavernous Sinus

**Figure 4:**
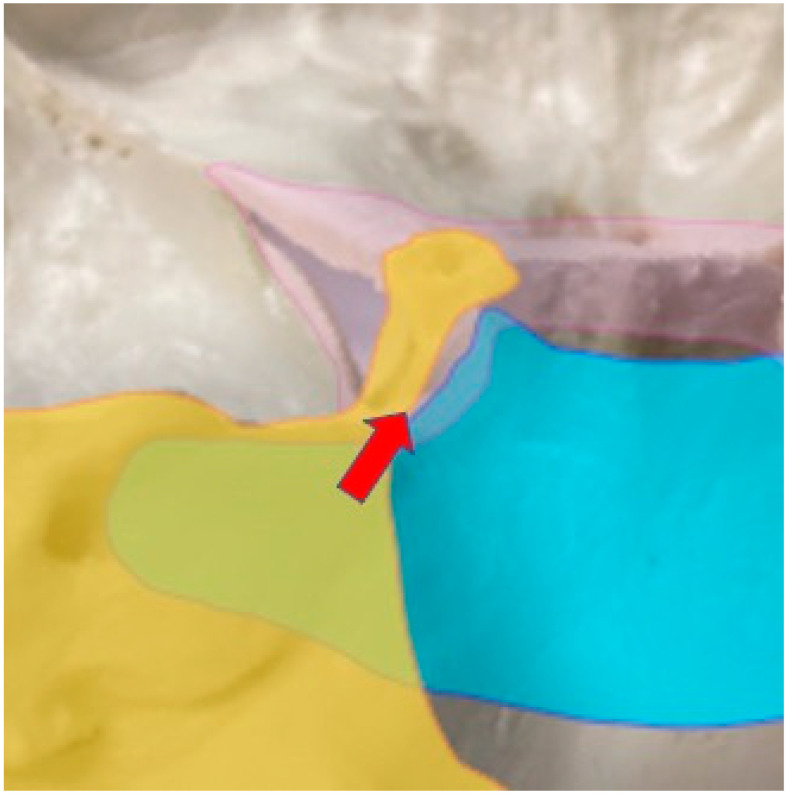
Image of the TA and DMA distribution around the cavernous sinus. Cavernous sinus(pink). The superolateral part of the posterior cavernous sinus seems to be supplied by the TA (yellow). The inferomedial part of the posterior cavernous sinus seems to be supplied by the DMA (blue). Cranial nerves are spaced between these vessels (arrow).

Among skull base meningiomas, cavernous sinus meningioma is extremely challenging in terms of tumor resection, and the treatment strategy for this area also remains controversial [13,18,19,20]. In addition to clinical and surgical difficulties, the anatomical features also make it difficult to clearly define the tumor origin between the cavernous sinus and the sphenoid ridge, petrous apex, upper clivus, and tentorium. This is because the dura is continuous around these areas. This article classified the cavernous sinus into Area 1 because the upper clivus and the dorsum sellae are continuous, so discriminating a clear tumor border between the cavernous sinus and the upper clivus is very difficult on MRI. If we try to classify these areas into different parts, the results would be very subjective, and there would be more concerns about misclassification. On the other hand, the tentorium and the cavernous sinus are slightly easier to differentiate because the clivus and the petrous bone offer good landmarks for estimating origins. Although our classification gives physicians a relatively easy method for judging borders, there might be some argument regarding this classification from a physiological perspective, because the feeding artery to the cavernous sinus is from the tentorial artery [5.19]. In this study, the DMA showed a strong correlation with Area 1, but for 66.7% of patients (48/72), the DMA was not the feeding artery even when the attachment was in Area 1. This suggests that some part of the CS might be supplied from arteries other than the DMA; this is likely to be the TA. Judging from our analysis and anatomical distributions from a previous report [21,22], we speculate that if the main origin of the tumor is the superolateral part of the cavernous sinus, there is a possibility that the tumor is fed by the TA, and if the inferomedial part of the cavernous sinus is involved, the DMA is believed to supply the tumor. In other words, when the TA is clearly observed as a feeding artery, the main attachment of the tumor might be at the superolateral part of the CS (Figure 4). This type of tumor is defined as petrotentorial meningioma, and cranial nerves might form the medial border to the tumor. If the feeding artery is the DMA, the tumor origin can be inferred to be at the inferomedial wall of the cavernous sinus, and this type of tumor is defined as petroclival meningioma. Cranial nerves might then form the lateral part of the tumor. However, there is ample room for further detailed classification and examination of feeding arteries for the cavernous sinus.

## 5. Limitations

Although we analyzed a relatively large number of cases with the rare disease skull base meningioma, we cannot rule out the possibility of selection or information biases since this was a single-center analysis. Furthermore, only patients who underwent angiography were included in this study. Anther limitation is the difficulty in determining the accurate quantity of perfusion from the feeding artery by the DSA. Although an approximate extent of blood supply can be assumed based on the running course of the vessels and tumor stain, it is difficult to clearly determine how much of the blood is supplied by the feeding artery, especially in case of meningioma with multiple feeders. If such quantitative analysis can be achieved in future, assessment of the feeding artery has the potential to predict the effect of tumor embolization or which artery has priority to be embolized first. In the view of the surgical strategy, such assessment can provide information for vascular manipulation prioritization during the operation.

## 6. Conclusions

In skull base meningiomas, cerebral angiography provides important anatomical information about tumor attachment. The DMA and Area 1, the APA and Area 2, and the TA and Area 3 are particularly highly correlated, and the PB of the MMA is correlated with Area 3. These feeding arteries are the essential feeders for each region of skull base meningioma. Evaluating and understanding these feeding arteries preoperatively can be helpful for determining adequate surgical strategies.

## Figures and Tables

**Table 1:** Summary of feeding arteries in skull base meningiomas.

Feeding Artery	Number of Arteries
Internal carotid artery	
Tentorial artery	62
Dorsal meningeal artery	45
Posterior ethmoidal artery	9
External carotid artery	
Middle meningeal artery	
Petrosal branch	44
Petrosquamous branch	3
Anterior branch	8
Recurrent meningeal artery	12
Accessory meningeal artery	15
Artery of foramen rotundum	18
Sphenopalatine artery	7
Middle deep temporal artery	5
Occipital artery	8
Ascending pharyngeal artery	41
Posterior auricular artery	3
Vertebral artery	
Posterior meningeal artery	6
Tentorial branch from PCA or SCA	9

**Table 2:** Correlation of feeding arteries and tumor attachment: multi/univariate analysis.

	Area 1	Area 2	Area 3
Dorsal meningeal artery	high/high	NS/NS	NS/NS
Ascending pharyngeal artery	NS/moderate	high/high	NS/NS
Tentorial artery	NS/moderate	NS/NS	high/high
MMA petrosal branch	NS/NS	NS/NS	moderate/high

MMA: middle meningeal artery; high: *p* < 0.001 moderate: *p* < 0.05. NS: Not Significant.

**Table 3:** Associations between tumor attachment to Area 1 and the dorsal meningeal artery.

		Attachment to Area 1		
		+	−	Total
DMA	+	44	1	45
	−	48	24	72
	total	92	25	117

**Table 4:** Association between tumor attachment to Area 2 and the ascending pharyngeal artery (APA).

		Attachment to Area 2		
		+	−	Total
APA	+	37	4	41
	−	13	63	76
	total	50	67	117

**Table 5:** Association between tumor attachment to Area 3 and tentorial artery (TA).

		Attachment to Area 3		
		+	−	Total
TA	+	53	9	62
	−	14	41	55
	total	67	50	117

**Table 6:** Association between tumor attachment to Area 3 and the petrosal branch (PB) of the MMA.

		Attachment to Area 3		
		+	−	Total
PB	+	35	9	44
	−	32	41	73
	total	67	50	117

MMA: middle meningeal artery.

## Data Availability

The data presented in this study are available upon request from the corresponding author. The data are not publicly available due to data privacy.
